# Concurrent Akt, ERK1/2 and AMPK Activation by Obestatin Inhibits Apoptotic Signaling Cascades on Nutrient-Deprived PC12 Cells

**DOI:** 10.1007/s10571-020-01025-8

**Published:** 2021-01-05

**Authors:** Agustín Sánchez-Temprano, José Luis Relova, Jesús P. Camiña, Yolanda Pazos

**Affiliations:** 1 Laboratorio de Patología Digestiva, Instituto de Investigación Sanitaria de Santiago (IDIS), Complejo Hospitalario Universitario de Santiago (CHUS), Servicio Gallego de Salud (SERGAS), 15706 Santiago de Compostela, Spain; 2 Laboratorio de Endocrinología Celular, IDIS, CHUS, SERGAS, 15706 Santiago de Compostela, Spain; 3 Departamento de Fisiología, Universidad de Santiago de Compostela (USC), 15706 Santiago de Compostela, Spain

**Keywords:** Obestatin, GPR39, Neuroprotection, Akt, AMPK, ERK1/2

## Abstract

Targeting apoptosis in the ischemic penumbra is a rational therapeutic approach for restricting cerebral infarct volume after clinical stroke. The present work explored the capability of the obestatin peptide, as a novel approach to inhibit apoptotic signaling cascades on PC12 cells. According to the results, obestatin treatment significantly reduced nutrient deprivation-induced apoptotic cell death. The protective effects were related to the regulation of the anti-apoptotic protein, BCL-2, and the apoptotic protein caspase-3. This encompasses the control of apoptosis by the interplay between Akt, ERK1/2 and AMPK signaling pathways. The activation of Akt and AMPK was concomitant with the phosphorylation of their downstream targets, GSK3 and ACC, respectively. Besides, obestatin also causes FoxO1 nuclear export supporting the prevention of the apoptosome formation. The concurrent activation of Akt and AMPK by obestatin via the GPR39 receptor, supports a role for this system in the balance concerning the catabolic and the anabolic signaling to sustain cellular function and viability. Furthermore, these results provide both an insight into how the obestatin/GPR39 system regulates anti-apoptotic pathways, and a framework for ascertaining how this system can be optimally targeted in treatment of brain cell death after stroke.

## Introduction

After a focal ischemic stroke, the reduced blood flow causes lesions in the nucleus of the brain tissue leading to the death of the necrotic cells. (Lo et al. 2003; Broughton et al. 2009; Moskowitz et al. 2010). Surrounding this necrotic core, a less affected area of tissue known as ischemic penumbra, is still metabolically active, although it remains functionally silent due to decreased blood flow. Apoptosis is the most relevant mechanism of cell death in the penumbra in which both caspase-dependent and caspase-independent mechanisms have been described (Yuan and Yankner 2000; Uzdensky 2019). In this peri-infarct margin, cells die more slowly as the penumbra collapses and the lesion expands over time, which potentially allows its recovery in a short time frame after the stroke. (Broughton et al. 2009; Moskowitz et al. 2010). Indeed, this therapeutic window seems to correspond temporarily with the initiation of caspase activation, whose inhibitors, administered up to that point of the protease activation, are able to attenuate ischemic brain injury and neurological function (Moskowitz et al. 2010). Therefore, targeting apoptotic-like mechanisms provide an opportunity for therapeutic focused to limit cerebral infarct volume (Dalkara and Moskowitz 2011).

Our previous works on the obestatin/GPR39 system demonstrated its key role in the regulation of skeletal muscle repair, supporting it as a promising therapeutic target (Gurriarán-Rodríguez et al. 2012, 2015; Santos-Zas et al. 2016). The 23-amino acid peptide obestatin, originated from the precursor polypeptide preproghrelin, controls de myogenic program driving anabolic processes within the activation of the G protein‐coupled receptor, GPR39 (Santos-Zas et al. 2016). Obestatin signaling regulates multiple steps of the myogenesis machinery by using G protein- and β‐arrestin dependent pathways with ERK1/2 and AKT activation being the primary effectors, respectively (Santos-Zas et al. 2016). More importantly, obestatin/GPR39 system transforms the identity of the muscle fiber, modifying the skeletal muscle phenotype into an oxidative profile (Santos-Zas et al. 2017) and counteracts deregulations in proteostasis by restoring efficient basal homoeostasis (Cid-Diaz et al. 2017).

AKT signaling plays important roles in neuronal survival, thereby influencing brain function with implications in a diverse set of neurological disorders (Manning and Toker 2017; Brunet et al. 2001). Thus, it is possible to speculate about the neuroprotective effect of the obestatin/GPR39 system as regulator of AKT-driven anabolic program. To test this hypothesis, we evaluated the activation of obestatin signaling in PC12 cells in which apoptotic cell death was induced by nutrient deprivation. In this work, we describe a potential neuroprotective function for the obestatin/GPR39 system by inhibition of the apoptotic-like mechanisms outstanding in ischemic stroke.

## Materials and Methods

### Materials

All the materials used in this section are listed in Table 1.

**Table 1:** Materials used in the analyses performed in this work

Antibodies	Use	Conc.	Details
Obestatin	IHC	1:100	Abcam Cat# ab41704, RRID:AB_776891
GPR39	IHC	1:500	Abcam Cat# ab39227, RRID:AB_941685
ß-actin	WB	1:1000	Santa Cruz Biotechnology Cat# sc-7210, RRID:AB_2223518
BCL-2	WB	RTU	Dako, RRID:SCR_013530; Cat#IS614
Caspase 3	WB	1:1000	Abcam Cat# ab2302, RRID:AB_302962
pERK1/2(T202/Y204)	WB	1:1000	Cell Signaling Technology Cat# 9101, RRID:AB_331646
ERK1/2	WB	1:1000	Cell Signaling Technology Cat# 9102, RRID:AB_330744
pGSK3α/ß(S21/9)	WB	1:1000	Cell Signaling Technology Cat# 9331, RRID:AB_329830
GSK3α/ß	WB	1:1000	Santa Cruz Biotechnology Cat# sc-56913, RRID:AB_783600
pAKT(S473)	WB	1:1000	Cell Signaling Technology Cat# 9271, RRID:AB_329825
Akt	WB	1:1000	Cell Signaling Technology Cat# 9272, RRID:AB_329827
pAMPKα (T172)	WB	1:1000	Cell Signaling Technology Cat# 2535, RRID:AB_331250
AMPKα	WB	1:1000	Cell Signaling Technology Cat# 2603, RRID:AB_490795
pACC(S79)	WB	1:1000	Cell Signaling Technology Cat# 3661, RRID:AB_330337
α-tubulin	WB	1:1000	Santa Cruz Biotechnology Cat# sc-5546, RRID:AB_635001
FoxO1	IF	1:100	Cell Signaling Technology Cat# 2880, RRID:AB_2106495

Materials	Details
Rat/mouse obestatin	BCN peptides, Cat#OBE-15X1
RPMI 1640	Lonza, RRID:SCR_000377, Cat#12-167F
Foetal bovine serum, FBS	Thermo Fisher Scientific, RRID:SCR_008452, Cat#11521851
Horse serum, HS	Lonza, RRID:SCR_000377, Cat#14-427F
Penicillin–Streptomycin-Glutamine, GPS	Thermo Fisher Scientific, RRID:SCR_008452, Cat#10378016
EnVision™ FLEX antibody diluent	Dako, RRID:SCR_013530, Cat#DM830
EnVision™ FLEX peroxidase blocking regent	Dako, RRID:SCR_013530, Cat#SM801
3,3′-Diaminobenzidine-tetrahydrochloride, DAB	Dako, RRID:SCR_013530, Cat#SM803
Cell Counting Kit-8, CCK8	Dojindo Laboratories, Cat#CK04
Hank’s balanced salt solution, HBSS	Thermo Fisher Scientific, RRID:SCR_008452, Cat#24020-091
Propidium iodide, PI	Invitrogen Antibodies, RRID:SCR_008410, Cat#P3566
4′,6‐Diamidino‐2‐phenylindole, DAPI	Invitrogen Antibodies, RRID:SCR_008410, Cat#D1306
QuantiPro™ BCA	Sigma-Aldrich, RRID:SCR_008988, Cat#QPBCA
ECL Western Blotting Substrate	Thermo Fisher Scientific, RRID:SCR_008452, Cat#32106

*Conc.* concentration, *IF* immunofluorescence, *IHC* immunohistochemistry, *WB* Western blot, *RTU* ready to use

### Cell Culture

PC-12 cells (ATCC Cat# CRL-1721, RRID:CVCL_0481) were cultured on collagen-I-coated plates with RPMI 1640 containing 10% FBS, 5% heat inactivated HS and 1% GPS. Cells were kept at 37 ºC in a humidified atmosphere containing 5% CO_2_.

### Immunocytochemistry

PC12 cells cultured on collagen-I-coated coverslips (5000 cells/cm^2^) were fixed in 96% (v/v) ethanol. Cell samples were consecutively incubated with: (1) primary antibody (Table 1) in EnVision™ FLEX antibody diluent; (2) EnVision™ FLEX peroxidase blocking regent used as the detection system; and (3) diaminobenzidine (DAB+) chromogenic substrate system. Harris’ hematoxylin was used to counterstain the samples.

### Proliferation Assay

PC12 cells were seeded on collagen type I-coated 96-well plates (5000 cells/cm^2^) using DMEM with 5% FBS. Upon 24 h, serum was withdrawn for 18 h and the cells were treated or not with obestatin (50–200 nM) for 48 h. The cell proliferation was evaluated using CCK8 according to the manufacturer's instructions.

### Cell Viability Assays

PC12 cells were seeded as described above at a ratio of 30,000 cells/cm^2^ and serum was withdrawn for 16 h. Cells were then treated with obestatin (200 nM) in HBSS for 6 h. Viability was detected using the CCK-8 kit, according to the manufacturer’s instructions.

### Propidium Iodide Staining

PC12 cells were cultured (5000 cells/cm^2^) as indicated above and treated or not with 200 nM obestatin. Then, the cells were fixed (MeOH), washed with citrate buffer [0.03 M sodium citrate (pH 7.0)] and then incubated with PI. The cell nuclei were counterstained with DAPI. Confocal images were obtained with a Leica TCS SPE (Leica TCS SPE, RRID:SCR_002140) version 8 confocal microscope. The M2 Manders coefficient (Manders et al. 1993) was quantified by the Fiji image processing software (Fiji, RRID:SCR_002285) version 2.1.0/1.53c.

### Immunoblot Analysis

The protein was obtained after lysing the cells in ice cold radioimmunoprecipitation assay (RIPA) buffer as previously described (Cid-Diaz et al. 2017). The QuantiPro™ BCA assay kit was used to quantify the protein concentration. The proteins were resolved by SDS-PAGE and subsequently transferred onto nitrocellulose membranes. Then, the membranes were incubated with the corresponding antibodies (Table 1). The bands were detected by ECL and digitalized using a ChemiDoc MP system (BioRad Laboratories; RRID:SCR_008426). Finally, the protein bands were analyzed by the Fiji image processing software.

### Immunofluorescence

PC12 cells were seeded as indicated for the cell viability assay. Upon serum withdrawn for 16 h, the cells were then treated or not with 200 nM obestatin in HBSS (6 h). Immunofluorescence was carried out as previously described (Cid-Diaz et al. 2017). Anti-FoxO1 antibody was used as primary antibody, using DAPI to visualize the cell nuclei. Confocal images were obtained with a Leica confocal microscope. Pearson coefficient was quantified by Fiji image software.

### Data Analysis

The data were analyzed with the GraphPad Prism processing program (GraphPad Prism, RRID:SCR_002798) version 8.2.1. The results are displayed as mean ± SEM. The statistical significance among groups was determined with Mann–Whitney test or one-way ANOVA post hoc test (Fisher's Least Significant Difference). The asterisks * or **, represent a *P* value of < 0.05 or < 0.01, respectively.

## Results

### Obestatin Enhances PC12 Cell Proliferation

**Fig. 1:**
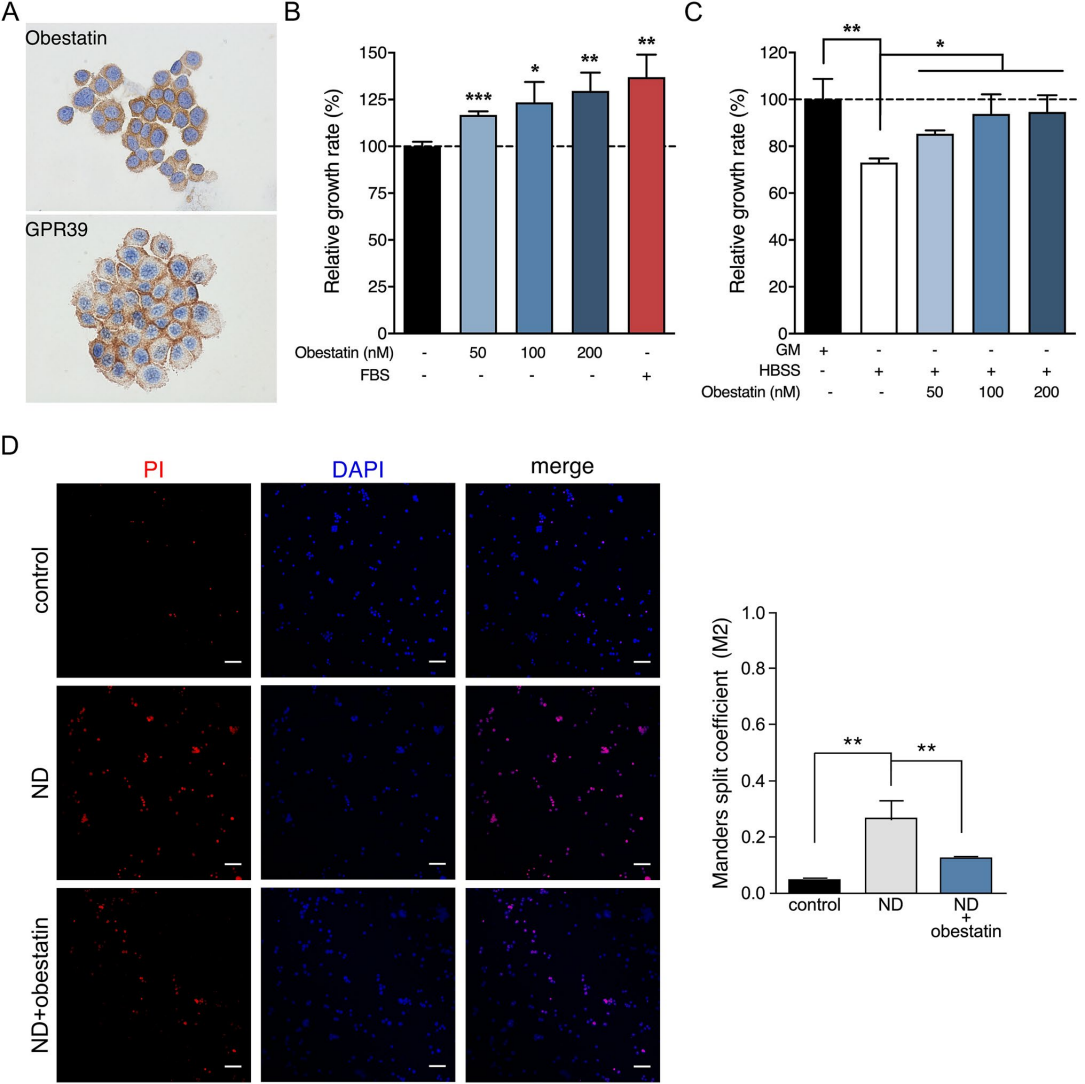
**a** Immunocytochemical expression of obestatin and GPR39 (upper and lower panel, respectively) in PC12 cells. **b** Dose–response effect of obestatin on PC12 cell proliferation determined by CCK8 assay (*n* = 8 per group). **c** Dose–response effect of obestatin on cell viability evaluated by CCK8 assay in nutrient-deprived PC12 cells (*n* = 6 per group). **d** Effect of obestatin (200 nM) on cell apoptosis evaluated by PI staining in nutrient-deprived PC12 cells. *Left panel*, representative images of PC12 cells stained with PI (red). Nucleus were counterstained with DAPI (blue). Right panel, quantification of Manders split coefficient (M2). Data were expressed as mean ± SEM. Asterisk (*, **) indicates *P* < 0.05 and *P* < 0.01 (ANOVA, Fisher’s LSD)

The capability of obestatin/GPR39 system as a switch between cell survival and death was addressed in PC12 cells, a cell line widely used to study neuroprotective effects of drugs (de los Rios et al. 2018). We first evaluated the presence of obestatin and GPR39 in PC12 cells by immunocytochemistry. Intense GPR39 positivity was observed associated to the plasma membrane whereas an also strong but more diffuse immunostaining was detected for obestatin in the same area (Fig. 1a). We then defined a dose-rate effect for obestatin (50–200 nM) on proliferation of PC12 cell showing its maximal effect at 200 nM (Fig. 1b).

### Obestatin Signaling Protects PC12 Cells from Nutrient Deprivation-Induced Apoptotic Cell Death

**Fig. 2:**
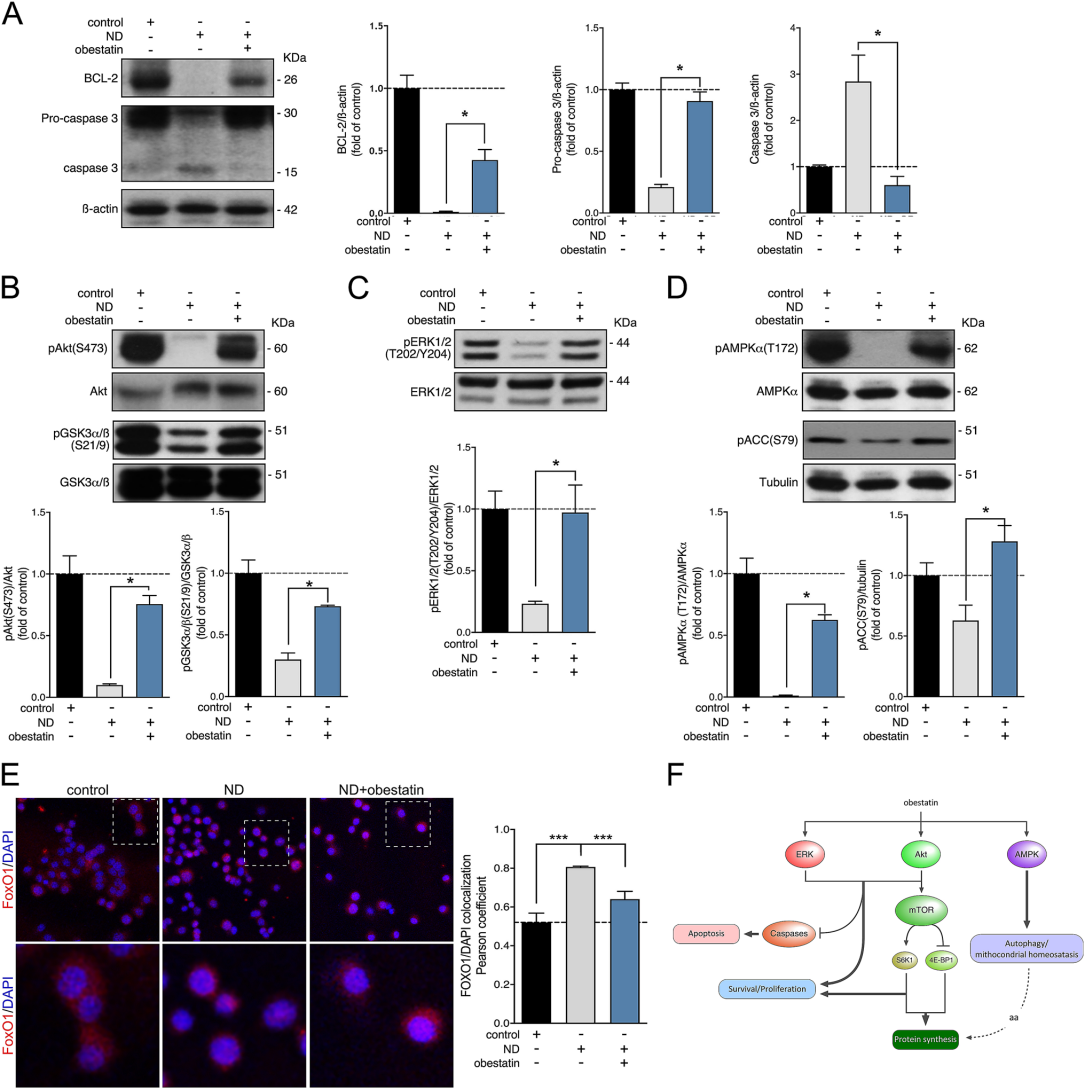
Analysis by immunoblots of BCL-2 and caspase 3 (**a**), phosphorylation of Akt at S473 [pAkt(S473)] and GSK3a/b at S21/9 [pGSK3 a/b(S21/9)] (**b**), phosphorylation of ERK1/2 at T202/Y204 [pERK1/2(T202/Y204)] (**c**), and phosphorylation of AMPKa at T172 [p AMPKa(T172)] and ACC at S79 [pACC(S79)] (**d**), in control, nutrient-deprived (ND) and obestatin-treated (200 nM) nutrient-deprived (ND + OB) PC12 cells. **e** *Left panel*, immunofluorescence detection of FoxO1 in control, ND and ND + OB PC12 cells. The cellular localization was determined using fluorescence microscopy using DAPI to label cell nuclei. *Right panel*, quantification of Pearson coefficient. Data were expressed as mean ± SEM (*n* = 3 per group). (***, *P* < 0.001; ANOVA, Fisher’s LSD). **f** Schematic diagram of the crosstalk between the catabolic and the anabolic processes activated by obestatin/GPR39 signaling. From **a** to **d**, data were expressed as mean ± SEM (*n* = 6 per group). Immunoblots are representative of the mean value. Asterisk (*) denotes *P* < 0.05 (Mann–Whitney test)

Incubation of PC12 cells for 6 h under nutrient deprivation conditions decreased cell viability, as compared to the control cultures (Fig. 1c). Co-incubation of the PC12-deprived cells with a range of obestatin concentrations (50–200 nM) reversed this effect (Fig. 1c). Remarkably, the cell death of PC12-deprived cells under obestatin administration (200 nM), estimated by Manders correlation coefficient between DAPI and PI (M2), was decreased to a similar level of those PC12 cells without nutrient deprivation conditions (Fig. 1d). At molecular level, this result was in line with the changes observed in the caspase 3 apoptosis indicator (D’Amelio et al. 2010). Nutrient deprivation conditions promoted caspase 3 activity by a synchronized decline of pro-caspase 3 (mw ≅ 30 KDa) and an enhancing of caspase 3 levels (mw ≅ 15 KDa) in PC12 cells, whereas obestatin treatment (200 nM) clearly reversed the deprivation-induced change (Fig. 2a). Furthermore, obestatin-treated PC12 cells exhibited significant increase of BCL-2, an anti-apoptotic protein (Fig. 2a) (Czabotar et al. 2014). The concurrent caspase 3 downregulation and BCL-2 upregulation pointed to the involvement of the growth promoting Akt-signaling pathway (Manning and Toker 2017). In fact, Akt activation was observed as an increasing in the phosphorylation of its S473 regulatory residue [pAkt(S473)], after obestatin treatment (200 nM) compared to PC12-nutrient deprived cells (Fig. 2b). The up‐regulation of Akt activity was concomitant with an increase in GSK3 phosphorylation at S21/9 (Fig. 2b). Furthermore, obestatin markedly promoted ERK1/2 at T202/Y204 on PC12-deprived cells (Fig. 2c). Strikingly, AMPK activation, evaluated by its phosphorylation at the T172 residue, augmented in response to obestatin related to untreated PC12-deprived cells (Fig. 2d). This correlated with increased phosphorylation of ACC, a downstream AMPK substrate, at S79 (Fig. 2d). For these targets, obestatin treatment achieved equivalent levels to those reached in control PC12 cells. Finally, the endogenous FoxO1 localization was studied by immunofluoresecence, and the nuclear to cytoplasmic fluorescence ratio was calculated by overlapping coefficient according to Pearson. Figure 2e shows that Fox1 was located mainly in the nucleus in PC12-deprived cells; however, obestatin treatment causes Fox1 translocation to cytoplasm, a similar pattern to that observed in untreated control cells. Similar results were observed when analyzing the Pearson coefficient, detecting a remarkable reduction for this coefficient in obestatin-treated cells compared to PC12-deprived cells (Fig. 2e). The obtained results demonstrate the function of the obestatin/GPR39 system in the activation of the antiapoptotic program, a function that is finely regulated by the interaction of the Akt, ERK1/2- and AMPK-signaling axes.

## Discussion

The characteristics of obestatin signaling to prevent cell death offers a promising strategy to address nerve damage and slow down any degeneration of nervous system. The main finding of this work is the neuroprotective action exerted by the obestatin/GPR39 system, since obestatin considerably reduces the cell death induced by nutrients deficiency in PC12 cells. These capabilities are due to a fine tuning of the Akt signaling pathway as well as its downstream target, BCL-2, in concert with the inhibition of cleaved-caspase 3 expression. This further includes the simultaneous control of the ERK1/2 pathway encompassing a series of effector proteins, which eventually tip the outcome in favor of cell survival. Strikingly, AMPK responds to obestatin, and its activation was independent of the Akt activity. Thus, cellular homeostasis of metabolism and growth is exquisitely controlled by the obestatin/GPR39 system by coordination of Akt, ERK1/2 and AMPK signaling cascades.

Akt and ERK1/2 transduce survival signals in response to obestatin (Santo-Zas et al. 2016). It has been described that ERK1/2 activation causes inhibition of caspase 9 activity through its phosphorylation and, as a consequence, the inhibition of caspase 3 processing, which ultimately inhibits apoptosis (Allan et al. 2003). On the other hand, the activation of Akt is associated with the restraint of the apoptotic process at different stages of its signaling cascade, including the braking of the sensors and effectors of the mitochondrial apoptotic pathway at molecular, transcriptional and metabolic levels (Manning and Toker 2017). Akt regulates FoxO1 transcriptional activity by site-specific phosphorylation and nuclear export (Matsuzaki et al. 2003; Aoki et al. 2004). Indeed, FoxO1 inactivation up-regulates the antiapoptotic protein BCL2, which avoids cytochrome c output from the mitochondria, the apoptosome formation and downstream caspase (Czabotar et al. 2014; Wang et al. 2018). Therefore, both mechanisms demonstrate overlapping features that impact on effector caspases, i.e. caspase-3, that target substrates to dismantle the cell (D'Amelio et al. 2010).

Paradoxically, this study reveals concurrent activation of Akt and AMPK by the obestatin/GPR39 system, two pathways typically considered as antagonistic signaling events. The coupling between the anabolic and catabolic functions appears to sustain core anabolic functions required for cell viability. Consistent with this, previous studies described active catabolic machinery in the context of active anabolic signaling in which augment their respective functions and facilitate the mass synthesis of secretory proteins (Narita et al. 2011; Kaur and Debnath 2015; Dalle-Pezze et al. 2016). Thus, this study provides an unexpected cellular task for the obestatin/GPR39 system in the regulation of catabolic processes enabling cells to maintain the correct functioning and the health of the cells (Fig. 2f). Further insight will be required to clarify the precise molecular features involved in this unconventional pathway.

At this point, we conclude that the concurrent activation of catabolic and anabolic pathways by the obestatin/GPR39 system promotes cell survival while inhibiting death effectors. These findings suggest that proper co-manipulation of these signaling pathways denote a potential strategy to limit neuronal damage.
